# Managing Patients With Myelofibrosis in the Era of Janus Kinase Inhibitors

**Published:** 2015-11-01

**Authors:** Emily A. Knight, Sylvia Osunsuyi-Fagbemi, Jessica Neely

**Affiliations:** 1Mayo Clinic, Scottsdale, Arizona; 2Winship Cancer Institute at Emory University, Atlanta, Georgia

**Managing Patients With Myelofibrosis in the Era of Janus Kinase Inhibitors**

A continuing education article for nurse practitioners, physician assistants, clinical nurse specialists, advanced degree nurses, oncology and hematology nurses, pharmacists, and physicians.

**Release date:** November 23, 2015

**Expiration date:** November 23, 2016

**Expected time to complete this activity as designed:** 1.0 hours

**Meniscus Educational Institute**

3131 Princeton Pike,

Building 1, Suite 205A

Lawrenceville, NJ 08648

Voice: 609-246-5000

Fax: 609-449-7969

E-mail: lrubin@meniscusedu.com

**Journal of the Advanced Practitioner in Oncology**

94 N. Woodhull Road

Huntington, NY 11743

Voice: 631-692-0800

Fax: 631-692-0805

E-mail: claudine@harborsidepress.com

© *2015, Meniscus Educational Institute. All rights reserved.*

## Faculty

**Emily A. Knight, RN, BSN, OCN®,** Mayo Clinic, Scottsdale, Arizona

**Sylvia Osunsuyi-Fagbemi, RN, BSN, OCN®,** Winship Cancer Institute at Emory University, Atlanta, Georgia

**Jessica Neely, PA-C, MMSC,** Winship Cancer Institute at Emory University, Atlanta, Georgia

## Activity Rationale and Purpose

Myelofibrosis (MF) is a rare, chronic myeloproliferative neoplasm. Oncology advanced practitioners (APs), including nurse practitioners, oncology nurses, pharmacists, and physician assistants, who are caring for patients with MF play important roles in patient education and the management of patient expectations. Vigilance in the monitoring of disease progression, treatment response, and adverse effects is crucial for delivering successful therapy. Because of the rarity of MF, many APs are unfamiliar with the new classification of drugs for the treatment of MF, Janus kinase inhibitors. Ruxolitinib is the first FDA approved drug in this category. The purpose of this article is to help equip oncology APs with the knowledge they need in order to educate their MF patients, to help them to remain on therapy, and ultimately, to maximize the overall treatment benefit.

## Intended Audience

The activity’s target audience will consist of nurse practitioners, physician assistants, clinical nurse specialists, advanced degree nurses, oncology and hematology nurses, pharmacists, and physicians.

## Learning Objectives

After completing this educational activity, participants should be able to:

1. Describe the pathophysiology of myelofibrosis and the role of JAK2 mutations
2. Discuss the clinical manifestations of myelofibrosis and their etiology
3. Explain the rationale for ruxolitinib dose adjustments based on platelet count and/or response to treatment
4. Identify strategies to enhance patient outcomes through patient education, assistance, monitoring, and optimizing treatment adherence

## Continuing Education

**Statement of Credit—Participants who successfully complete this activity (including the submission of the post-test and evaluation form) will receive a statement of credit.**

**Physicians.** The Meniscus Educational Institute is accredited by the Accreditation Council for Continuing Medical Education (ACCME) to provide continuing medical education for physicians.

The Meniscus Educational Institute designates this journal article for a maximum of 1.0 *AMA PRA Category 1 Credits*™. Physicians should claim only the credit commensurate with the extent of their participation in the activity.

**Nurses.** This activity for 1.0 contact hour is provided by the Meniscus Educational Institute.

The Meniscus Educational Institute is accredited as a provider of continuing nursing education by the American Nurses Credentialing Center’s Commission on Accreditation.

Provider approved by the California Board of Registered Nursing, Provider No. 13164, for 1.0 contact hour.

**Pharmacists.** The knowledge-based accredited education lectures are intended for pharmacists involved in the care of cancer patients. This educational activity is sponsored by the Meniscus Educational Institute.

The Meniscus Educational Institute is accredited by the Accreditation Council for Pharmacy Education (ACPE) as a provider of continuing pharmacy education. The ACPE Universal Activity Number assigned to this program, for 1.0 contact hour, is 0429-9999-15-017-H01-P.

## Financial Disclosures

All individuals in positions to control the content of this program (eg, planners, faculty, content reviewers) are expected to disclose all financial relationships with commercial interests that may have a direct bearing on the subject matter of this continuing education activity. Meniscus Educational Institute has identified and resolved all conflicts of interest in accordance with the MEI policies and procedures. Participants have the responsibility to assess the impact (if any) of the disclosed information on the educational value of the activity.

**Faculty**

**Emily A. Knight, RN, BSN, OCN®,** has nothing to disclose.

**Sylvia Osunsuyi-Fagbemi, RN, BSN, OCN®,** has nothing to disclose.

**Jessica Neely, PA-C, MMSC,** has nothing to disclose.

**Lead Nurse Planner**

**Wendy J. Smith, ACNP, AOCN®,** has nothing to disclose.

**Planners**

**Jeannine Coronna** has nothing to disclose.

**Claudine Kiffer** has nothing to disclose.

**Terry Logan, CHCP,** has nothing to disclose.

**Pamela Hallquist Viale, RN, MS, CNS, ANP,** has nothing to disclose.

**Lynn Rubin** has nothing to disclose.

**Content Reviewers**

**Glenn Bingle, MD, PhD, FACP,** has nothing to disclose.

**Christopher J. Campen, PharmD, BCPS, BCOP,** has served on the advisory board for Taiho Oncology.

**Karen Abbas, MS, RN, AOCN®,** has nothing to disclose.

**Wendy J. Smith, ACNP, AOCN®,** has nothing to disclose.

## Disclaimer

This activity has been designed to provide continuing education that is focused on specific objectives. In selecting educational activities, clinicians should pay special attention to the relevance of those objectives and the application to their particular needs. The intent of all Meniscus Educational Institute educational opportunities is to provide learning that will improve patient care. Clinicians are encouraged to reflect on this activity and its applicability to their own patient population.

The opinions expressed in this activity are those of the faculty and reviewers and do not represent an endorsement by Meniscus Educational Institute of any specific therapeutics or approaches to diagnosis or patient management.

## Product Disclosure

This educational activity may contain discussion of published as well as investigational uses of agents that are not approved by the US Food and Drug Administration. For additional information about approved uses, including approved indications, contraindications, and warnings, please refer to the prescribing information for each product.

## How to Earn Credit

To access the learning assessment and evaluation form online, visit www.meniscusce.com

## ABSTRACT

Myelofibrosis, a rare, chronic myeloproliferative neoplasm associated with progressive bone marrow fibrosis and extramedullary hematopoiesis, is a life-shortening disease with a heterogeneous clinical presentation that poses significant challenges for the advanced practitioner in oncology in patient assessment and management. Common clinical manifestations of myelofibrosis are splenomegaly, various spleen-related and constitutional symptoms, and anemia. Optimal management includes regular spleen size assessment by palpation as well as monitoring symptoms and quality of life with validated instruments to evaluate therapeutic response and disease progression. The Janus kinase 1 (JAK1)/JAK2 inhibitor ruxolitinib, which has been shown to provide effective and lasting spleen size reduction and symptom mitigation as well as a survival advantage compared with placebo and best available therapy in randomized controlled clinical trials, has been approved for the treatment of myelofibrosis in more than 80 countries worldwide. However, ruxolitinib is associated with dose-dependent cytopenias, particularly thrombocytopenia, attributable to its mechanism of action; therefore, blood cell counts should be monitored and dose adjusted as necessary. Through continual patient education and support, advanced practitioners in oncology can help patients to remain on therapy and ultimately maximize overall treatment benefit.

## ARTICLE

Myelofibrosis (MF) is a rare, chronic myeloproliferative neoplasm (MPN) characterized by progressive bone marrow fibrosis and extramedullary hematopoiesis. Myelofibrosis affects mostly elderly patients (Cervantes et al., 2009; Emanuel et al., 2012), and recent estimates suggest an annual incidence rate of 2 to 3 cases per 100,000 persons in the United States (Mehta, Wang, Iqbal, & Mesa, 2014).

Myelofibrosis is a heterogeneous disease with a diverse etiology (Mesa et al., 2011) and pathogenesis (Lundberg et al., 2014), resulting in a highly variable clinical presentation (Emanuel et al., 2012; Mitra et al., 2013) and prognosis (Cervantes et al., 2009; Vannucchi et al., 2013b). The most common clinical manifestations include splenomegaly, a variety of MF-related symptoms, and anemia. Many of the profound signs of MF are associated with shortened survival (Cervantes et al., 2009; Gangat et al., 2011; Passamonti et al., 2010), and disease-related symptoms may greatly diminish a patient’s quality of life (QOL; Mesa et al., 2007; Mesa et al., 2009b; Mesa et al., 2013b).

Allogeneic hematopoietic stem cell transplantation remains the only potentially curative treatment. However, because of high risks for treatment failure and treatment-related mortality, the procedure is generally reserved for select younger patients who have a poor prognosis (e.g., those who have a high risk of transformation to acute myeloid leukemia) but otherwise are healthy enough to withstand the procedure (Babushok & Hexner, 2014).

Until November 2011, when the Janus kinase 1 (JAK1)/JAK2 inhibitor ruxolitinib (Jakafi) became the first drug approved by the US Food and Drug Administration (FDA) for the treatment of patients with intermediate- or high-risk MF, pharmacologic management of patients with MF was limited to a multimodal approach aimed at symptom mitigation. Most of these conventional therapies tended to be poorly or only temporarily effective and/or had dose-limiting adverse effects such as severe myelosuppression (Mesa, 2013; Vannucchi, 2011). In December 2014, ruxolitinib also gained FDA approval for the treatment of patients with polycythemia vera who have had an inadequate response to or are intolerant of hydroxyurea (Incyte Corporation, 2014).

The treatment paradigm in MF fundamentally changed with the positive results of two pivotal phase III clinical trials (the Controlled Myelofibrosis Study With Oral JAK Inhibitor Treatment [COMFORT]), which evaluated the efficacy and safety of ruxolitinib in patients with intermediate-2 or high-risk MF and baseline platelet counts of at least 100 × 10^9^/L compared with placebo (COMFORT-I; Verstovsek et al., 2012b) or best available therapy (BAT; COMFORT-II; Harrison et al., 2012).

In these studies, ruxolitinib provided rapid and lasting reduction in splenomegaly and MF-associated symptom burden in most patients and improved QOL measures compared with placebo or BAT (Cervantes et al., 2013; Harrison et al., 2012; Harrison et al., 2013; Mesa et al., 2013a; Verstovsek et al., 2012b; Verstovsek et al., 2013c; Verstovsek et al., 2015). In addition, there is now compelling evidence from multiple analyses involving these and other studies that ruxolitinib reduces the risk of death by 30% to 50% compared with placebo or BAT (Cervantes et al., 2013; Kantarjian et al., 2013; Passamonti et al., 2014; Vannucchi et al., 2013a; Verstovsek et al., 2012a; Verstovsek et al., 2013c), suggesting that ruxolitinib may alter the natural history of the disease.

Oncology advanced practitioners (APs), including nurse practitioners, clinical nurse specialists, and physician assistants, who are caring for patients with MF play important roles in patient education and the management of patient expectations. Furthermore, vigilance in the monitoring of disease progression, treatment response, and adverse effects is crucial for delivering successful therapy. Given the variable clinical presentation of MF and the ubiquitous comorbidities in this mostly elderly patient population, the tasks required of APs are challenging. Moreover, many APs may lack experience in the care of patients with MF because of the rarity of the disease.

In this article, we summarize the disease characteristics of MF and the treatment effects of ruxolitinib and discuss the role of oncology APs in ensuring maximal treatment benefit for patients receiving ruxolitinib therapy.

## PATHOGENESIS, CLINICAL PRESENTATION, AND PROGNOSIS

**Dysregulated JAK-STAT Signaling: Key Feature of Pathobiology**

Myelofibrosis may originate as primary myelofibrosis (PMF) or result from myelofibrotic transformation of essential thrombocythemia (ET) or polycythemia vera (PV) to post-ET or post-PV MF, respectively (Barosi et al., 2008; Mesa et al., 2011). Although PMF, ET, and PV are related, they are clinically and histomorphologically distinct Philadelphia chromosome–negative MPNs (Vardiman et al., 2009). However, PMF, post-ET MF, and post-PV MF are phenotypically similar, as a patient’s signs and symptoms are dominated by the effects of progressive bone marrow fibrosis, regardless of the underlying MPN (Barosi et al., 2008; Mesa et al., 2011).

The molecular and cellular events leading to the development of bone marrow fibrosis in patients with MPNs are incompletely understood but likely constitute a reaction by bone marrow stromal cells to malignant hematopoietic stem cell clones mediated by fibrogenic and proinflammatory cytokines (Hasselbalch, 2013; Le Bousse-Kerdilès, Martyré, & Samson, 2008). As bone marrow fibrosis progresses, the increasingly diminished hematopoietic capacity of the bone marrow leads to cytopenias (particularly anemia) and/or organomegaly (particularly splenomegaly), which is due to extramedullary hematopoiesis.

A key pathobiologic feature shared by PMF, ET, and PV is overactive signaling through the JAK-STAT (signal transducers and activators of transcription) pathway (Rampal et al., 2014), which is essential for the control of definitive hematopoiesis (Vainchenker, Dusa, & Constantinescu, 2008).

Several mutations have been linked to MPN pathogenesis, including the gain-of-function mutation *JAK2V617F*, which results in constitutive JAK2 activation in hematopoietic stem cells and is found in at least 95% of patients with PV and the majority of those with PMF or ET (Cross, 2011; Klampfl et al., 2013; Lundberg et al., 2014; Nangalia et al., 2013; Payzin et al., 2014). JAK-STAT pathway overactivation in MPNs also occurs in patients who do not have the *JAK2V617F* mutation (Rampal et al., 2014). Moreover, dysregulated JAK-STAT signaling not only underlies the neoplastic proliferation and impaired maturation of hematopoietic stem cells, but it is also responsible for high levels of circulating inflammatory cytokines, which have been linked to the high burden of constitutional symptoms in MF (Dueck et al., 2013; Squires et al., 2013).

**Clinical Presentation**

The most common clinical manifestation of MF is splenomegaly, which may affect more than 80% of patients with PMF (Cervantes et al., 2009) and can be highly symptomatic (Table 1). In a large retrospective analysis, approximately one-third of patients with PMF had marked splenomegaly, defined as a palpable spleen length > 10 cm (Emanuel et al., 2012). Splenomegaly may cause symptoms of variable severity, ranging from early satiety and abdominal discomfort to severe abdominal pain, and potentially serious complications, such as portal hypertension and splenic infarcts (Mesa, 2009; Mughal, Vaddi, Sarlis, & Verstovsek, 2014; Tefferi, 2000). In addition to the spleen, other organs, particularly the liver, may be affected by extramedullary hematopoiesis, and the occurrence of hepatomegaly is a major concern of palliative splenectomy (Benjamini, Jain, Estrov, Kantarjian, & Verstovsek, 2012; Mesa & Tefferi, 2001; Mughal et al., 2014).

**Table 1:**
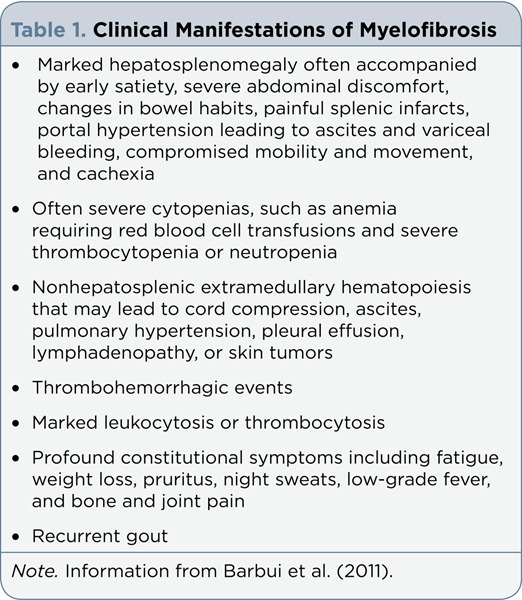
Clinical Manifestations of Myelofibrosis

Other common disease manifestations include anemia, which occurs in approximately 50% of patients with PMF (Emanuel et al., 2012), as well as debilitating constitutional symptoms arising from chronic cytokine-driven hypercatabolism (e.g., weight loss, fatigue) and inflammation (e.g., fever, pruritus, night sweats; Geyer et al., 2014; Mesa, 2013; Mesa et al., 2007; Mesa et al., 2013b). Although the nature and severity of MF-related symptoms may vary substantially (Geyer et al., 2014), overall symptom burden is a major contributor to the often poor QOL among patients with MF (Mesa et al., 2007; Mesa et al., 2013b).

**Prognosis**

Patients with MF have a reduced life expectancy and an increased risk of secondary acute myeloid leukemia, which is associated with a median survival of less than 3 months (Mesa et al., 2005). Although up to 30% of PMF-related deaths result from leukemic transformation (Vannucchi, 2011), most patients with MF die from a variety of complications related to disease progression, including but not limited to bone marrow failure, organ failure, thrombohemorrhagic events, infections, and portal hypertension (Cervantes et al., 2009; Verstovsek et al., 2013c).

To improve the prognostication of patients with PMF at diagnosis, the International Working Group for Myelofibrosis Research and Treatment (IWG-MRT) developed a risk-stratification system based on the number of validated risk factors present: the International Prognostic Scoring System (Cervantes et al., 2009). Although it has not been validated outside of PMF, this scoring system has also been used for the risk stratification of patients with post-ET or post-PV MF in clinical trials (Harrison et al., 2012; Verstovsek et al., 2012b).

Similar models with additional risk factors, the Dynamic International Prognostic Scoring System (DIPSS) and the DIPSS Plus can be used to determine a patient’s prognosis at any time of the disease course, independent of the time of diagnosis or treatment initiation (Table 2; Gangat et al., 2011; Passamonti et al., 2010). Patients are classified as low-, intermediate-1-, intermediate-2-, or high-risk, with median life expectancies ranging from 15 years for low-risk patients to less than 2 years for high-risk patients (Gangat et al., 2011). Apart from these classic prognostic scoring systems, cytokine levels (Tefferi et al., 2011), the presence and number of specific mutations (Lundberg et al., 2014; Tefferi et al., 2014; Vannucchi et al., 2013b), bone marrow fibrosis grade (Gianelli et al., 2012; Lekovic et al., 2014), splenomegaly (Mesa et al., 2015; Vannucchi et al., 2013a), and comorbidities (Lekovic et al., 2014) may have prognostic significance.

**Table 2:**
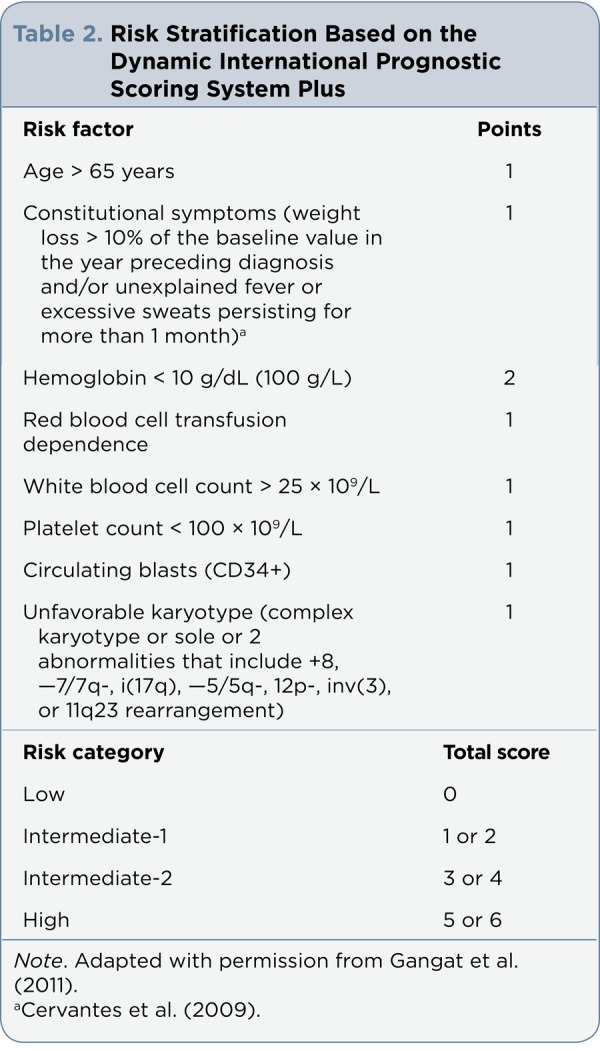
Risk Stratification Based on the Dynamic International Prognostic Scoring System Plus

## EFFICACY AND SAFETY OF RUXOLITINIB

**Efficacy**

The primary efficacy endpoint in the COMFORT-I and COMFORT-II studies was the proportion of patients with a ≥ 35% reduction in spleen volume at 24 and 48 weeks, respectively (Harrison et al., 2012; Verstovsek et al., 2012b). In COMFORT-I, 41.9% vs. 0.7% of patients in the ruxolitinib and placebo groups (*p* < .001), respectively, met the primary endpoint (Verstovsek et al., 2012b). In COMFORT-II, 28% of the patients in the ruxolitinib group and no patients in the BAT group (*p* < .001) met the primary endpoint (Harrison et al., 2012). In COMFORT-I, patients with a ≥ 35% spleen volume reduction at any time during the study follow-up had a 53% probability of maintaining this level of reduction for at least 132 weeks (Verstovsek et al., 2015).

Similarly, patients in the ruxolitinib arm of COMFORT-II who achieved a ≥ 35% spleen volume reduction had a 50% probability of maintaining this response at week 144 (Cervantes et al., 2013). Mean percentage reduction in spleen volume from baseline in the ruxolitinib arm of COMFORT-I was 31.6% at week 24 and 34.1% at week 144 (Verstovsek et al., 2012b; Verstovsek et al., 2015). In contrast, patients who received placebo had a mean increase in spleen volume of 8.1% at week 24 (Verstovsek et al., 2012b), and all patients had discontinued study participation or crossed over to ruxolitinib (as permitted by the study design) within 3 months of the primary analysis, with a median time to crossover of 41.1 weeks (Verstovsek et al., 2015).

Results of the COMFORT studies also demonstrated that ruxolitinib therapy was associated with marked improvements in symptoms and other patient-reported outcomes (Harrison et al., 2013; Mesa et al., 2013a). In COMFORT-I, 45.9% of patients randomized to receive ruxolitinib (vs. 5.3% in the placebo group, *p* < .001) had a ≥ 50% improvement in total symptom score (TSS) at week 24 (as assessed by the modified Myelofibrosis Symptom Assessment Form [MFSAF] v2.0; Verstovsek et al., 2012b), including improvements in abdominal discomfort, pain under the left ribs, early satiety, night sweats, itching, bone/muscle pain, and inactivity (Mesa et al., 2013a).

In addition, whereas global health status/QOL and all five functional domains (physical, cognitive, role, emotional, social) of the European Organization for Research and Treatment of Cancer Quality-of-Life Questionnaire-Core 30 (EORTC QLQ-C30) worsened in the placebo group, significant improvements were found in the ruxolitinib group among patients who experienced a ≥ 50% reduction in TSS (Mesa et al., 2013a Notably, ruxolitinib treatment was also associated with a significant improvement in global health status as well as physical, social, and role functioning among patients with < 50% reduction in TSS.

Symptom improvement tended to be most pronounced in patients who also attained a ≥ 35% reduction in spleen volume but also was observed in those with a 10% to < 35% spleen volume reduction (Mesa et al., 2013a). Weight and cholesterol levels also improved with ruxolitinib therapy in COMFORT-I (Mesa et al., 2015). Consistent with its efficacy in symptom mitigation, ruxolitinib reduced circulating levels of proinflammatory cytokines in the COMFORT studies, particularly tumor necrosis factor–alpha and interleukin-6 (Harrison et al., 2012; Verstovsek et al., 2012b), which have been linked to cancer-related cachexia (Figueras et al., 2005; Iwase, Murakami, Saito, & Nakagawa, 2004; Scott, McMillan, Crilly, McArdle, & Milroy, 1996).

In COMFORT-II, EORTC QLQ-C30 scores for global health status/QOL as well as physical, role, and social functioning improved significantly in the ruxolitinib groups vs. the BAT group (Harrison et al., 2013). In addition, dyspnea, insomnia, pain, diarrhea, fatigue, and appetite loss (assessed with the EORTC QLQ-C30) improved with ruxolitinib vs. BAT (Harrison et al., 2013). Ruxolitinib, compared with BAT, also had a favorable effect on the scores of the 15-item lymphoma subscale, which assesses pain, swelling, fever, night sweats, itching, trouble sleeping, fatigue, weight loss, loss of appetite, and other patient concerns (Harrison et al., 2013).

Overall, long-term results of the COMFORT studies suggest that ruxolitinib therapy is associated with a survival advantage compared with placebo (Verstovsek et al., 2013c; Verstovsek et al., 2015) or BAT (Cervantes et al., 2013). Given the prognostic impact of constitutional symptoms and nutritional status (Cervantes et al., 2009; Mesa et al., 2015; Sulai et al., 2012), this may be explained in part by the reduction in symptom burden and overall improvement of metabolic status with ruxolitinib. Furthermore, there is emerging evidence that ruxolitinib may provide long-term improvement in or stabilization of bone marrow fibrosis in some patients (Kvasnicka et al., 2013a, 2013b).

Results of other small studies and case series further suggest that the benefits of ruxolitinib may extend to reduction of hepatomegaly in patients with MF (Benjamini et al., 2012), the improvement of hematologic and cardiac parameters in patients with MF-related pulmonary hypertension (Tabarroki et al., 2014), and the improvement of signs and symptoms of splanchnic vein thrombosis in patients with MPNs (Pieri et al., 2013).

**Safety**

Given the critical role of JAK2 signaling in hematopoiesis, cytopenias are expected consequences of the mechanism of action of ruxolitinib. Not surprisingly, anemia and thrombocytopenia were the most common treatment-related adverse events in the COMFORT trials (Harrison et al., 2012; Verstovsek et al., 2012b).

In COMFORT-I, 45.2% and 12.9% of patients in the ruxolitinib group had grade 3 or 4 anemia and thrombocytopenia, respectively, compared with 19.2% and 1.3% in the placebo group (Verstovsek et al., 2012b). Grade 3 or 4 neutropenia was also more common with ruxolitinib (7.1%) than placebo (2.0%; Verstovsek et al., 2012b), whereas leukopenia was uncommon because most patients had elevated white blood cell counts at treatment initiation (Verstovsek et al., 2013b).

Despite the occurrence of dose-dependent anemia and thrombocytopenia with ruxolitinib, these events were rarely a cause of treatment discontinuation (Harrison et al., 2012; Verstovsek et al., 2012b) and could generally be managed with dose adjustments, brief treatment interruptions, or red blood cell transfusions in patients with anemia (Mesa & Cortes, 2013). An analysis of COMFORT-I data showed that hemoglobin levels tended to return to just below baseline values and platelet counts tended to stabilize after initial decreases during the first 2 to 3 months of therapy (Verstovsek et al., 2013b). As a result, the incidence of new-onset anemia and thrombocytopenia decreased substantially after the first 3 months of therapy (Mesa & Cortes, 2013; Verstovsek et al., 2013b). Furthermore, the majority of patients had final titrated (average daily) doses of 10 mg twice daily or higher by the end of week 24 and that these doses were associated with optimal or near-optimal efficacy in terms of spleen volume reduction and symptom relief (see Figure; Mesa & Cortes, 2013; Verstovsek et al., 2013b).

**Figure:**
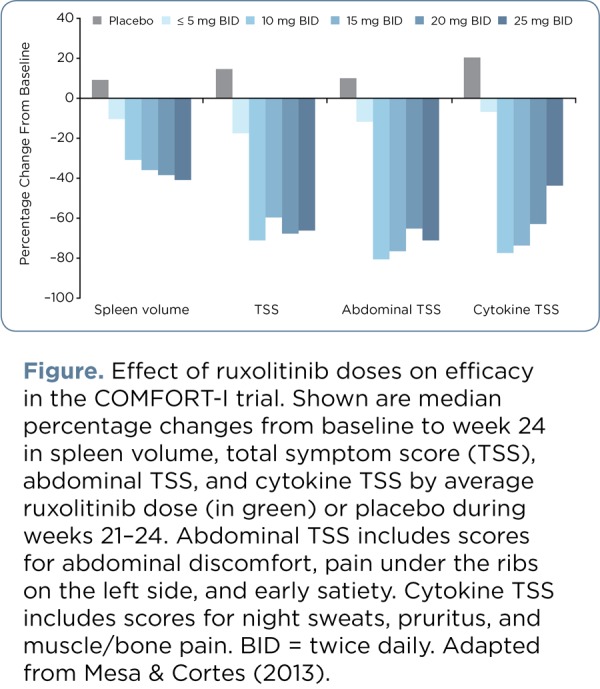
Effect of ruxolitinib doses on efficacy

Nonhematologic adverse effects of ruxolitinib are generally grade 1 or 2 and consist primarily of gastrointestinal disturbances (i.e., diarrhea, headache, dizziness, ecchymosis; Verstovsek et al., 2012b), with the latter potentially related to the presence of thrombocytopenia. Long-term therapy with ruxolitinib in the COMFORT studies was not associated with any increase in new-onset adverse events, and no unexpected safety signals were observed (Cervantes et al., 2013; Verstovsek et al., 2013c; Verstovsek et al., 2015).

## CONVENTIONAL AND EXPERIMENTAL THERAPIES

Although standard therapy for MF-related symptoms overall seems to be no more effective than placebo (Mesa et al., 2014), traditional therapies such as interferon-α, hydroxyurea, oral alkylating agents (i.e., melphalan, busulfan), immunomodulatory agents (i.e., thalidomide [Thalomid], lenalidomide [Revlimid], pomalidomide [Pomalyst]), hypomethylating agents (e.g., azacitidine, decitabine), corticosteroids, androgenic steroids (e,g., danazol), and erythropoiesis-stimulating agents (Mesa, 2013; Mitra et al., 2013) may still be valuable for the treatment of patients who require additional palliative care for symptoms not treatable with ruxolitinib (e.g., anemia) or who do not tolerate or respond to ruxolitinib.

For example, patients with anemia may benefit from anti-anemia therapy with danazol, erythropoiesis-stimulating agents, or immunomodulatory agents (Odenike, 2013). Although immunomodulatory agents may be useful for some patients with MF and anemia, their utility as monotherapy appears to be limited by modest activity and/or poor tolerability (Begna et al., 2012; Burgstaller et al., 2013; Daver et al., 2013, 2014; Odenike, 2013). Combinations of ruxolitinib with danazol, pomalidomide, and lenalidomide are currently evaluated in clinical trials (Yacoub, Odenike, & Verstovsek, 2014).

One retrospective study of 62 patients with MF suggested that long-term therapy with recombinant interferon-α may be effective in patients with early-stage disease (Ianotto et al., 2013). Spleen, symptom, and anemia response per 2006 IWG-MRT consensus criteria was observed in 46.5%, 82.1%, and 72% of evaluable patients, respectively. In addition, normalization of corresponding blood cell counts occurred in all patients with leukocytosis, leukopenia, or thrombocytosis. However, palpable spleen length > 6 cm was associated with treatment failure, and time to best response varied from 4 to 9 months (Ianotto et al., 2013). Based on the limited available information, recombinant interferon-α may have a role in the treatment of patients with substantial residual hematopoietic function (Odenike, 2013) but is not recommended for patients with advanced MF or marked splenomegaly.

For patients who respond to JAK inhibitor therapy but do not tolerate ruxolitinib, enrollment in a clinical trial of an experimental JAK inhibitor with a different tolerability profile, including potentially less myelosuppression, e.g., pacritinib (Komrokji et al., 2015; Verstovsek et al., 2013a) or momelotinib (Pardanani et al., 2013, 2013b), may be considered. However, the presumed benefits of pacritinib and momelotinib in terms of increased hematologic tolerability still require confirmation in large randomized controlled trials. In addition, they may come with a trade-off in the form of nonhematologic safety concerns.

In a small phase II study (N = 35) of pacritinib in patients with intermediate- or high-risk MF, including 43% with platelet counts < 100 × 10^9^/L at baseline, only 2 patients discontinued pacritinib for thrombocytopenia. However, gastrointestinal adverse effects were very common (diarrhea, 77.1%; nausea, 45.7%; Komrokji et al., 2015).

In a phase I/II study of momelotinib, 23 of 33 patients (70%) requiring red blood cell transfusions during the month preceding study entry were transfusion-free for at least 12 weeks during the study (Pardanani et al., 2013b), suggesting that momelotinib may be capable of inducing anemia responses. Yet a recent analysis revealed that 44% of patients treated with momelotinib developed persistent peripheral neuropathy after a median time to onset of 32 weeks (Abdelrahman et al., 2015).

Patients with an inadequate response to JAK inhibitor therapy may be eligible for clinical trials that include other classes of compounds. They include histone deacetylase inhibitors, kinase inhibitors not targeting JAKs, and antifibrotic or antianemic agents. Several agents are currently being evaluated for combination therapy with ruxolitinib in clinical trials; they include panobinostat (Farydak; a histone deacetylase inhibitor), RPM-151 (recombinant human pentraxin-2), the antifibrotic monoclonal antibody simtuzumab, the hedgehog inhibitor LDE225 (erismodegib), and the PI3K inhibitor buparlisib (BKM120; Odenike, 2013; Yacoub et al., 2014). Panobinostat has shown promising spleen and anemia responses in phase I research, including complete resolution of splenomegaly in three of five patients evaluable for response assessment, and resolution of bone marrow fibrosis in one patient (Mascarenhas et al., 2013).

Splenic irradiation may provide transient pain relief in patients with highly symptomatic splenomegaly refractory to medical therapy (Barbui et al., 2011; Mesa, 2009). Palliative splenectomy may be an option for select patients with symptomatic splenomegaly refractory to medical therapy, portal hypertension, and/or spleen-related anemia; however, the procedure is associated with high rates of potentially serious complications and does not improve outcomes (Mesa, 2013; Mesa & Tefferi, 2001).

## OPTIMIZING PATIENT CARE

**Initial Assessment and Continual Disease Monitoring**

At initial presentation, a standard holistic approach should be taken to determine each patient’s medical history, including prior diagnosis of PV or ET, previous hemorrhagic and thrombotic events, previous and current infections, previous diagnoses or signs of cardiovascular and other morbidities, and overall nutritional and performance status. Definitive diagnosis of MF is based on clinical signs and symptoms, as well as testing for relevant genetic markers and evaluation of bone marrow biopsies (Barosi et al., 2008; Vardiman et al., 2009).

Assessment of splenomegaly, hepatomegaly, symptom burden, karyotype, and blood cell counts is vital in aiding diagnosis, establishing risk, and aiding therapeutic decision-making. Palpation is appropriate for the assessment of splenomegaly in clinical practice, although more precise methods for quantitative spleen response evaluation, such as magnetic resonance imaging and computed tomography, are recommended for clinical trials (Pardanani et al., 2013b).

For patients who frequently require blood transfusions because of anemia, testing for iron overload is also important. In COMFORT-I, up to 40% of patients in the ruxolitinib group required red blood cell transfusions in any given 4-week period during the first 24 weeks of therapy compared with approximately 20% to 30% of patients in the placebo group (Verstovsek et al., 2012b). However, the monthly rate of patients in the ruxolitinib group who required transfusions decreased over time and was generally around 30% after week 24 (Verstovsek et al., 2012b).

Given the heterogeneity of MF-related signs and symptoms and the high frequency of comorbidities, careful symptom evaluation and management are keys to successful care to ultimately improve QOL for this unique elderly patient population. A variety of patient-reported cancer assessment tools have been used in clinical trials to systematically assess symptom burden in affected patients, including the EORTC QLQ-C30, the Patient-Reported Outcomes Measurement Information System (PROMIS) fatigue scale, and the Functional Assessment of Cancer Therapy–Lymphoma (FACT–Lym) scale (Harrison et al., 2013; Mesa et al., 2013a). The PROMIS fatigue scale is valuable for the quantification of general fatigue (Garcia et al., 2007), which has been shown to have a profound negative effect on QOL in patients with MPNs (Mesa et al., 2007), whereas the EORTC QLQ-C30 may be used to assess overall health and performance status (Harrison et al., 2013; Mesa et al., 2013b).

Two recently developed instruments were designed specifically for the quantitative assessment of MF- and MPN-related symptom burden on a scale from 0 (absent) to 10 (worst imaginable): the MFSAF (Barbui et al., 2011; Mesa et al., 2009b) and the closely related Myeloproliferative Neoplasm Symptom Assessment Form (MPN-SAF) TSS (Emanuel et al., 2012; Scherber et al., 2011).

In COMFORT-I, an abbreviated version of the MFSAF was used to rate the severity of seven MF-related symptoms: night sweats, pruritus, abdominal discomfort, pain under the ribs (left side), early satiety, bone/muscle pain, and inactivity (Mesa et al., 2013a; Verstovsek et al., 2012b). The 10-item MPN-SAF TSS questionnaire in combination with a question from the MD Anderson Cancer Center Brief Fatigue Inventory (Table 3; Emanuel et al., 2012) is a convenient instrument for patient evaluation in clinical practice, both at screening and for monitoring treatment response. The MPN-SAF TSS strongly correlated with overall QOL and the EORTC QLC-C30 functional scales in an assessment of 1,408 patients with MPNs; the inclusion of a question on fatigue is an important aspect of this abbreviated scale, as it is one of the most common and most burdensome symptoms (Emanuel et al., 2012).

**Table 3:**
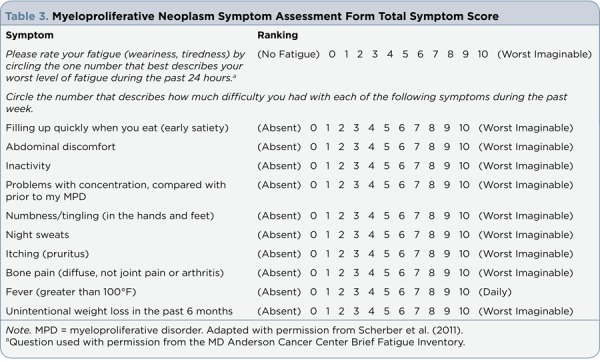
Myeloproliferative Neoplasm Symptom Assessment Form Total Symptom Score

These questionnaires not only are valuable tools for disease monitoring but also facilitate the communication between practitioner and patient regarding the patient’s symptoms. In addition to the discussion of common symptoms, specific follow-up questions should take into consideration the variability of the clinical manifestations of MF Table 1) and address the possibility of rare but serious complications arising from extramedullary hematopoiesis beyond hepatosplenomegaly, such as pulmonary or spinal embolism. This verbal follow-up is a critical part of patient evaluation, because many patients with MF may not be forthcoming about their symptoms. For example, patients who have symptoms for a long time may have developed coping mechanisms and therefore may not fully appreciate the burden or severity of their symptoms.

**Maximizing the Therapeutic Benefit of Ruxolitinib**

Treatment effects of ruxolitinib, including spleen and symptom responses and possible declines in platelet counts or hemoglobin values that may require dose adjustment or red blood cell transfusions, usually occur rapidly (i.e., within the first 8 to 12 weeks of therapy; Verstovsek et al., 2013b). Particularly close monitoring of disease parameters as well as monitoring of complete blood cell counts every 2 to 4 weeks is important during this initial treatment period, before blood cell counts have stabilized. After this time, monitoring should continue as clinically indicated (Incyte Corporation, 2014).

Patients responding to ruxolitinib should be encouraged to continue therapy, even if they require dose adjustments, to maintain therapeutic benefit. They should be advised that symptoms return to baseline levels within 1 week of discontinuing therapy. Thus, avoiding prolonged treatment interruptions or treatment discontinuations is key to maximizing the treatment benefit of ruxolitinib. The ruxolitinib prescribing information provides detailed recommendations for the monitoring of blood cell counts and the implementation of dose adjustments based on these counts (Incyte Corporation, 2014).

Per prescribing information, the recommended starting dose of ruxolitinib for MF depends on baseline platelet counts (i.e., 20 mg twice daily for a platelet count > 200 × 10^9^/L; 15 mg twice daily for a platelet count of 100 to 200 × 10^9^/L; and 5 mg twice daily for a platelet count of 50 to < 100 × 10^9^/L; Incyte Corporation, 2014). Complete blood cell counts, including platelet counts, should be determined before treatment initiation and every 2 to 4 weeks until doses are stabilized (Incyte Corporation, 2014).

If blood cell counts remain adequate (i.e., platelet count > 125 × 10^9^/L after 4 weeks of therapy and never below 100 × 10^9^/L, and the absolute neutrophil count is > 0.75 × 10^9^/L), doses may be increased to improve efficacy, to a maximum of 25 mg twice daily for patients with a starting platelet count ≥ 100 × 10^9^/L and 10 mg twice daily for patients with a starting platelet count of 50 to < 100 × 10^9^/L. Dose increases should be considered if reductions from baseline in palpable spleen length consistently are < 50% (Incyte Corporation, 2014).

Experience from COMFORT-I, which did not include patients with baseline platelet counts < 100 × 10^9^/L, suggests that the majority of patients with baseline platelet counts of 100 to 200 × 10^9^/L and a minority of those with baseline platelet counts > 200 × 10^9^/L may require dose reductions from their recommended starting dose, most often during the first 3 months of therapy (Verstovsek et al., 2013b). After initial dose titration, most patients in COMFORT-I achieved maintenance doses of 10 mg twice daily or higher, which have been associated with a clinically significant reduction in splenomegaly and symptom burden (Verstovsek et al., 2013b).

Thus, careful dose titration after treatment initiation guided by close monitoring of platelet counts is essential to avoid unnecessary drops in platelet counts, which may require prolonged interruptions or permanent discontinuation of therapy. The relationship between platelet counts and mandatory dose reductions (per prescribing information) for patients with baseline platelet counts ≥ 100 × 10^9^/L is summarized in Table 4.

**Table 4:**
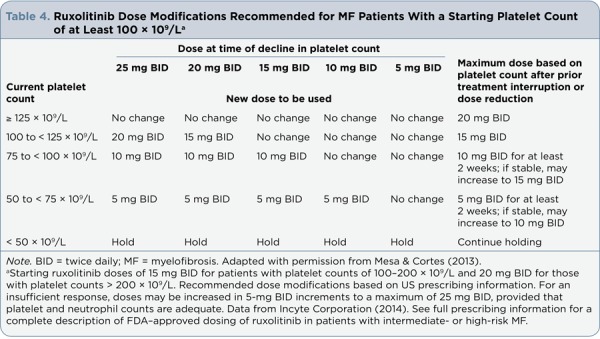
Ruxolitinib Dose Modifications Recommended for MF Patients With a Starting Platelet Count of at Least 100 × 10^9^/La

For patients with platelet counts of 50 to < 100 × 10^9^/L, uptitration from a starting dose of 5 mg twice daily in increments of 5 mg once daily is recommended, based on the interim results of a phase II study (Talpaz et al., 2013). For these patients, dosing modifications are necessary if platelet counts drop to 25 to < 35 × 10^9^/L, and dosing should be interrupted if platelet counts fall below 25 × 10^9^/L (Incyte Corporation, 2014).

During the initial dose-titration period, APs should also regularly monitor changes in palpable spleen length and symptoms to detect early signs of loss of efficacy caused by dose reductions or treatment interruptions. Patients and practitioners should be aware that reintroduction of ruxolitinib after a brief treatment interruption may restore efficacy (Gisslinger et al., 2014). Yet ruxolitinib should be discontinued in patients who experience no spleen size reduction or symptom improvement after 6 months of therapy (Incyte Corporation, 2014).

Monitoring of absolute neutrophil counts is important for the prevention or timely detection of severe treatment-related neutropenia (absolute neutrophil count < 0.5 × 10^9^/L), which can generally be reversed by treatment interruptions (Incyte Corporation, 2014). In COMFORT-I, decreases in neutrophil counts occurred primarily during the first 4 weeks of therapy (Verstovsek et al., 2013b). Therefore, absolute neutrophil counts should be monitored every 2 to 4 weeks after treatment initiation until counts have stabilized.

Although hemoglobin values should be monitored because disease- and treatment-related anemia is common in patients with myelofibrosis, anemia is not a contraindication for ruxolitinib and should be treated with red blood cell transfusions as necessary. Because many patients starting ruxolitinib experience often reversible decreases in hemoglobin within the first 8 weeks of therapy, hemoglobin values and red blood cell counts should be closely monitored, particularly in patients with or at risk for anemia. Prompt intervention to reverse exacerbating anemia should focus preferentially on red blood cell transfusion and antianemia therapy rather than dose reduction. Experience with the concomitant use of erythropoiesis-stimulating agents for the treatment of anemia in patients receiving ruxolitinib is limited, but one report suggested a potential benefit (McMullin et al., 2012).

Concomitant use of strong CYP3A4 inhibitors such as ketoconazole, indinavir, boceprevir, fluconazole (dose ≤ 200 mg), nefazodone, and grapefruit juice may greatly increase the serum concentrations of ruxolitinib and thus may require corresponding ruxolitinib dose reductions to minimize the risk of adverse events. Patients should not take fluconazole at doses > 200 mg daily concomitantly with ruxolitinib. Patients receiving the CYP3A4 inducer rifampin concomitantly with ruxolitinib should be monitored frequently, because rifampin may decrease the effective serum concentration of ruxolitinib (Incyte Corporation, 2014).

Patients with kidney or liver impairment require a modified starting dose of ruxolitinib. Patients with moderate or severe renal impairment (i.e., a creatinine clearance of 15 to 59 mL/min) should start ruxolitinib at a dose of 10 mg twice daily if the platelet count is 100 to 150 × 10^9^/L and at 5 mg daily if the platelet count is 50 to < 100 × 10^9^/L (Incyte Corporation, 2014). Ruxolitinib should not be used for patients with end-stage renal disease (creatinine clearance, < 15 mL/min) not requiring dialysis. Patients with any degree of hepatic impairment should start ruxolitinib therapy at a dose of 10 mg twice daily if the platelet count is 100 to 150 × 10^9^/L and at 5 mg daily if the platelet count is 50 to < 100 × 10^9^/L. Ruxolitinib should be avoided in patients with hepatic impairment or moderate or severe renal impairment if platelet counts are < 50 × 10^9^/L (Incyte Corporation, 2014).

As part of monitoring for adverse events, heightened vigilance is required in the treatment of patients with a potentially compromised immune system, as serious infections have occurred in patients treated with ruxolitinib, including progressive multifocal leukoencephalopathy (Wathes, Moule, & Milojkovic, 2013), hepatitis B reactivation (Caocci et al., 2014), pneumonia (Wysham, Sullivan, & Allada, 2013), and disseminated tuberculosis (Hopman, Lawrence, & Oh, 2014). Although these were isolated cases and the relationship with treatment remains unclear, immunosuppressive effects of ruxolitinib have been documented, including an in vitro and in vivo decrease in dendritic cell function in mice and an ex vivo decrease in T-regulatory cells in humans (Barosi et al., 2013; Heine et al., 2013; Massa, Rosti, Campanelli, Fois, & Barosi, 2014). Before the initiation of ruxolitinib therapy, patients should be evaluated for tuberculosis risk factors, such as prior residence in or travel to countries with a high prevalence of tuberculosis, close contact with a person with active tuberculosis, or a history of active or latent tuberculosis with potentially inadequate treatment. Those who test positive for active or latent infection should talk to their physician about the risks and benefits of ruxolitinib (Incyte Corporation, 2014).

## PATIENT EDUCATION

Patients who feel empowered by knowledge about their condition and treatment options tend to participate more actively in their own care. Patient education allows APs to forge a strong relationship with their patients, which may ultimately result in improved patient care. Advanced practitioners may act as a conduit of information between the patient and the interdisciplinary health-care team and may assume an important role in disseminating complex medical data to the patient in an easy-to-understand manner.

Practitioners participating in the care of patients who are treated with ruxolitinib are in a unique position to manage patients’ expectations regarding the possible benefits and side effects of this therapy, explain the clinical significance of test results, and educate patients about various aspect of their disease, including current risk status and possible symptoms and signs of disease progression. They should convey to their patients that ruxolitinib can result in durable benefits in spleen reduction and symptom score.

However, patients should also understand the risk and management of dose-dependent cytopenias. They should be advised that initial decreases in platelet counts and hemoglobin values are expected effects of ruxolitinib therapy; they do not represent worsening of the disease and are likely to improve or stabilize with appropriate management (Mesa & Cortes, 2013).

Patients should also be reminded regularly that prolonged treatment interruptions could result in the return of MF-related symptoms. Because marked worsening of returning symptoms after abrupt discontinuation of ruxolitinib therapy has been observed in a few isolated cases, gradual tapering of the dose should be considered if treatment is discontinued for reasons other than cytopenias (Incyte Corporation, 2014).

Optimal treatment adherence is critical to ensure the continued benefit of ruxolitinib therapy. Many patients with MF are elderly and have comorbidities that may require them to take multiple medications with potentially different schedules of administration. Advanced practitioners in oncology may assist patients in devising routines that minimize the risk of patients forgetting to take their medications. Patients should also understand that the treatment schedule for ruxolitinib during the day is flexible, and it can be taken with or without food. Patients who take additional medications need to be informed of potential drug-drug interactions.

Oncology advanced practitioners are in an ideal position to provide support for administrative procedures, such as obtaining insurance reimbursement (varies from state to state and by insurance carrier) and guiding patients to appropriate patient assistance programs (Table 5). These programs not only provide access to funds required to procure medical services, but also offer travel and lodging assistance and compliance aids to maximize the likelihood that patients adhere to treatment as prescribed.

**Table 5:**
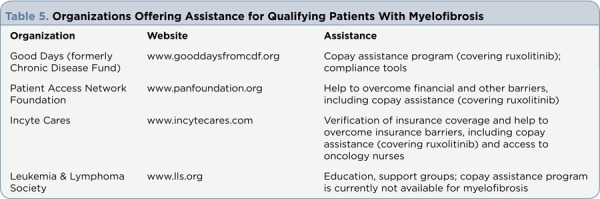
Organizations Offering Assistance for Qualifying Patients With Myelofibrosis

## CONCLUSIONS

Myelofibrosis is a rare, complex, chronic disease with a heterogeneous clinical presentation. It poses significant challenges for the oncology AP with respect to patient assessment and management. Optimal management requires consistent use of validated assessment tools to monitor the multiple symptoms throughout the disease course and to evaluate therapeutic response and disease progression. The JAK1/JAK2 inhibitor ruxolitinib has been shown to provide effective mitigation of signs and symptoms of MF as well as a survival advantage vs. placebo and BAT in clinical trials. Through continued patient education and support, APOs can help patients adhere to treatment schedules, optimize the management of treatment-related cytopenias to remain on therapy, and maximize overall treatment benefit.

**Acknowledgments**

Editorial support for the development of this article was provided by Malcolm Darkes, PhD, and Roland Tacke, PhD, of Evidence Scientific Solutions and was funded by Incyte Corporation.
